# Site-specific metabolic phenotypes in metastatic breast cancer

**DOI:** 10.1186/s12967-014-0354-3

**Published:** 2014-12-14

**Authors:** Hye Min Kim, Woo Hee Jung, Ja Seung Koo

**Affiliations:** Department of Pathology, Severance Hospital, Yonsei University College of Medicine, 50 Yonsei-ro, Seodaemun-gu, Seoul, 120-752 South Korea

**Keywords:** Breast, Metabolism, Metastasis

## Abstract

**Background:**

The purpose of this study was to examine the expression of metabolism-related proteins according to metastatic site in metastatic breast cancer and to assess the implication of site-specific differential expression.

**Methods:**

A tissue microarray containing 162 cases of metastatic breast cancer (52 lung metastasis, 47 bone metastasis, 39 brain metastasis, and 24 liver metastasis) was constructed. It was subject to immunohistochemical staining of the following proteins: *Glycolysis-related*: Glut-1, hexolinase II, carbonic anhydrase (CA) IX, and monocarboxylate transporter (MCT) 4; glutaminolysis-related: glutaminase (GLS) 1, glutamate dehydrogenase (GDH), and amino acid transporter (ASCT) 2; *mitochondrial metabolism-related*: ATP synthase, succinate dehydrogenase (SDH)A, and SDHB; and *serine/glycine metabolism related*: phosphoglycerate dehydrogenase (PHGDH), phosphoserine aminotransferase (PSAT), phosphoserine phosphatase (PSPH), glycine decarboxylase (GLDC), and serine hydroxymethyltransferase (SHMT).

**Results:**

The expression levels of glycolysis-related-proteins (Glut-1, hexokinase II, CAIX, and MCT4) differed according to metastatic site, with higher expression seen in the brain and lower expression in the bone and liver (p < 0.001, 0.001, 0.009, and <0.001, respectively). Differences in metabolic phenotype were analyzed according to metastasis site. Glycolysis type was most frequently encountered in the brain and lung (p < 0.001). In univariate analysis, the factors associated with shorter overall survival were CAIX positivity (p = 0.044), PSPH positivity (p = 0.045), and SHMT1 positivity (p = 0.002), as well as serine/glycine type (p = 0.041).

**Conclusions:**

Differences in metabolic features according to metastatic site were seen in metastatic breast cancer, with the glycolysis phenotype found predominantly in the brain and lung and the non-glycolysis phenotype in the bone and liver.

## Introduction

Breast cancer has a high morbidity and mortality, mainly because it can easily metastasize to distant organs. The main metastatic sites from breast cancer are the lung, bone, brain, and liver [1,2]. However, most studies have focused on bone and brain metastases [3-8]. The main mechanism of tumor metastasis is the reciprocal interaction between tumor cells and the host tissue, involving cell adhesion, proteolysis, invasion, and angiogenesis [2,9]. Because different cancers display distinct metastatic patterns, the seed and soil hypothesis has been proposed, which dictates that the specific tumor (seed) can survive only in specific visceral organs (soil) [10]. Accordingly, metastatic breast cancer cells show different characteristics according to the metastatic site. For example, brain metastasis is associated with young age, estrogen receptor (ER) negativity, prior lung metastasis, HER-2 overexpression, EGFR overexpression, and the basal subtype [5-7], while bone metastasis is associated with lower histologic grade, ER positivity, ER positivity/progesterone receptor (PR) negativity, strand growth pattern, and the presence of fibrotic foci in invasive ductal carcinoma [4,11,12]. Therefore, metastatic breast cancer is also likely to display distinct characteristics according to metastatic site.

According to the Warburg effect theory, while normal cells gain energy from oxidative phosphorylation, cancer cells obtain energy from glycolysis, making glycolysis an important component in cancer metabolism [13]. However, this theory cannot fully explain the energy usage of all cancer cells [14]. Glutamine and mitochondrial metabolism, along with glucose metabolism, are also important components in cancer cell metabolism. Tumor cells under active glycolysis have higher levels of glycolytic intermediates, and the metabolism of glycolytic intermediates has been recently shown to be involved in tumorigenesis. A representative metabolic pathway of glycolytic intermediates is the glycine and serine metabolic pathway [15-18], which has been recently studied as a new possible target for tumor therapy [19]. Targeted therapy can be used in metastatic cancer, as well as in primary cancer, making the identification of metabolic phenotypes in metastatic cancer clinically important. However, metastatic cancer displays distinct characteristics according to metastatic site, but the site-specific metabolic features have not yet been fully identified. The purpose of this study was to examine the expression of metabolism-related proteins according to their metastatic site in metastatic breast cancer and their implication.

## Materials and methods

### Patient selection

Patients with invasive primary breast cancer and metastasis to distant organs (lung, bone, brain, and liver) were selected from medical records of the Department of Pathology of Severance Hospital. Only patients with a diagnosis of invasive ductal carcinoma were included. In total, 162 cases were identified, and 49 cases were paired between primary cancer and metastatic cancer. All slides were reviewed, and pathologic diagnoses were approved by two pathologists (JSK and WHJ). Histological grade was assessed using the Nottingham grading system [20]. This study was approved by the Institutional Review Board (IRB) of Severance Hospital. Written informed consent was obtained from the patient for the publication of this report and any accompanying images.

### Tissue microarray

On H&E-stained slides of the tumors, a representative area was selected, and the corresponding spot was marked on the surface of the paraffin block. Using a biopsy needle, a 3-mm tissue core in the selected area was punched out and placed onto a 6 × 5 recipient block. Two tissue cores were extracted to minimize extraction bias. Each tissue core was assigned a unique tissue microarray location number that was linked to a database containing other clinicopathologic data.

### Immunohistochemistry (IHC)

The antibodies used for IHC in this study are shown in Table 1. Formalin-fixed, paraffin-embedded (FFPE) tissue samples were used as follows. Three-micron-thick slices from the FFPE tissue block were deparaffinized and rehydrated in xylene and alcohol solutions and stained using a Ventana Discovery XT automated stainer (Ventana Medical Systems, Tucson, AZ, USA). Antigen retrieval was performed with CC1 (Cell Conditioning 1) buffer (citrate buffer pH 6.0, Ventana Medical Systems). Appropriate positive and negative controls were used.

**Table 1:** **Clone, dilution, and source of antibodies used in this study**

**Antibody**	**Clone**	**Dilution**	**Source**
*Molecular subtype related*			
ER	SP1	1:100	Thermo Scientific, CA, USA
PR	PgR	1:50	DAKO, Denmark
HER-2	Polyclonal	1:1500	DAKO, Denmark
Ki-67	MIB-1	1:150	DAKO, Denmark
*Glycolysis related*			
Glut-1	SPM498	1:200	Abcam, Cambridge, UK
Hexokinase II	3D3	1:200	Abcam, Cambridge, UK
CAIX	Polyclonal	1:100	Abcam, Cambridge, UK
MCT4	Polyclonal	1:100	Santa Cruz, CA, USA
*Glutaminolysis related*			
GLS1	Polyclonal	1:50	Abcam, Cambridge, UK
GDH	Polyclonal	1:100	Abcam, Cambridge, UK
ASCT2	Polyclonal	1:100	Abcam, Cambridge, UK
*Mitochondrial related*			
ATP synthase	15H4C4	1:100	Abcam, Cambridge, UK
SDHA	2E3GC12FB2AE2	1:100	Abcam, Cambridge, UK
SDHB	21A11AE7	1:100	Abcam, Cambridge, UK
*Serine/glycine metabolism related*			
PHGDH	Polyclonal	1:100	Abcam, Cambridge, UK
PSPH	Polyclonal	1:100	Abcam, Cambridge, UK
PSAT1	Polyclonal	1:100	Abcam, Cambridge, UK
SHMT	Polyclonal	1:100	Abcam, Cambridge, UK
GLDC	Polyclonal	1:100	Abcam, Cambridge, UK

### Interpretation of immunohistochemical results

A cut-off value of 1% or more positively stained nuclei was used to define ER and AR positivity [21]. HER-2 staining was analyzed according to the American Society of Clinical Oncology (ASCO)/College of American Pathologists (CAP) guidelines using the following categories: 0 = no immunostaining; 1+ = weak incomplete membranous staining, less than 10% of tumor cells; 2+ = complete membranous staining, either uniform or weak in at least 10% of tumor cells; and 3+ = uniform intense membranous staining in at least 30% of tumor cells [22]. HER-2 staining was considered positive when strong (3+) membranous staining was observed whereas it was considered negative when none or weak (0 to 1+) staining was noted.

IHC result interpretation was based on the product of the proportion of stained cells and the immunhistochemical staining intensity. A product between 0 and 1 was regarded as negative, a product between 2 and 4 as low positive, and a product between 5 and 6 as high positive [23]. The proportion of stained cells was scored as 0 for negative, 1 for positive with less than 30%, and 2 for positive with greater than or equal to 30%. The staining intensity was scored as 0 for negative, 1 for weak, 2 for moderate, and 3 for strong. Ki-67 labeling index (LI) was defined as the percentage of positive cells in tumor cell nuclei.

### Tumor phenotype classification

In this study, breast cancer phenotypes were classified according to IHC results for ER, PR, HER-2, and Ki-67, as well as FISH results for HER-2 as follows [24]: *luminal A type*: ER or/and PR positive and HER-2 negative and Ki-67 LI <14%; *luminal B type*: (HER-2 negative) ER or/and PR positive and HER-2 negative and Ki-67 LI ≥14%, (HER-2 positive) ER or/and PR positive and HER-2 overexpressed or/and amplified; *HER-2 type*: ER and PR negative and HER-2 overexpressed or/and amplified; and *triple negative breast cancer* (*TNBC) type*: ER, PR, and HER-2 negative.

### Classification of tumor metabolic subtype

In this study, tumor metabolic subtypes were classified according to IHC results for metabolism-related proteins as follows: *Glycolysis type*: 3 or more positive glycolysis-related proteins [Glut-1, hexolinase II, carbonic anhydrase (CA) IX, and monocarboxylate transporter (MCT) 4]; *glutaminolysis type*: 2 or more positive glutaminolysis-related proteins [glutaminase (GLS) 1, glutamate dehydrogenase (GDH), and amino acid transporter (ASCT) 2]; *mitochondrial type*: 2 or more positive mitochondrial metabolism proteins [ATP synthase, succinate dehydrogenase (SDH)A, and SDHB]; and *serine/glycine type*: 3 or more positive serine/glycine metabolism-related proteins [phosphoglycerate dehydrogenase (PHGDH), phosphoserine aminotransferase (PSAT), phosphoserine phosphatase (PSPH), glycine decarboxylase (GLDC), serine hydroxymethyltransferase (SHMT)].

### Statistical analysis

Data were statistically processed using SPSS for Windows, version 12.0 (SPSS Inc., Chicago, IL, USA). Correlation analysis of immunostaining results between primary breast cancer and metastatic breast cancer was calculated by the McNemar test. Student’s *t* and Fisher’s exact tests were used to examine any differences in continuous and categorical variables, respectively. Corrected *p*-value and the Bonferroni method were used for multiple comparisons. Statistical significance was assumed when *P <*0.05. Kaplan-Meier survival curves and log-rank statistics were employed to evaluate time to tumor metastasis and time to survival. Multivariate regression analysis was performed using a Cox proportional hazards model.

## Results

### Baseline characteristics of patients

In a total of 162 cases, 52 (32.1%) had lung metastasis, 47 (29.0%) had bone metastasis, 30 (18.5%) had brain metastasis, and 24 (14.8%) had liver metastasis. The proportion of cases with ER positivity and PR positivity was higher among those with bone and liver metastases than in those with metastasis to other sites (p < 0.001), and HER-2 positivity was higher among cases of brain metastasis compared to other sites (p = 0.017). Furthermore, luminal A type tumors were more common among patients with bone and liver metastasis, while the proportion of tripe negative breast cancer (TNBC) was higher among cases of brain and lung metastasis (p < 0.001) (Table 2).

**Table 2:** **Basal clinicopathologic characteristics of patients with breast cancer metastasis according to metastatic site**

**Parameters**	**Total N = 162 (%)**	**Bone metastasis n = 47 (%)**	**Brain metastasis n = 39 (%)**	**Liver metastasis n = 24 (%)**	**Lung metastasis n = 52 (%)**	**p-value**
Age (yr, mean ± SD)	52.0 ± 10.5	52.3 ± 10.0	53.5 ± 11.7	54.2 ± 10.8	49.7 ± 9.5	0.221
ER						**<0.001**
Negative	69 (42.6)	8 (17.0)	26 (66.7)	6 (25.0)	29 (55.8)	
Positive	93 (57.4)	39 (83.0)	13 (33.3)	18 (75.0)	23 (44.2)	
PR						**<0.001**
Negative	109 (67.3)	23 (48.9)	38 (97.4)	12 (50.0)	36 (69.2)	
Positive	53 (32.7)	24 (51.1)	1 (2.6)	12 (50.0)	16 (30.8)	
HER-2						**0.017**
Negative	114 (70.4)	38 (80.9)	20 (51.3)	19 (79.2)	37 (71.2)	
Positive	48 (29.6)	9 (19.1)	19 (48.7)	5 (20.8)	15 (28.8)	
Molecular subtypes						**<0.001**
Luminal A	67 (41.4)	33 (70.2)	4 (10.3)	15 (62.5)	15 (28.8)	
Luminal B	27 (16.7)	7 (14.9)	9 (23.1)	3 (12.5)	8 (15.4)	
HER-2	30 (18.5)	5 (10.6)	12 (30.8)	3 (12.5)	10 (19.2)	
TNBC	38 (23.5)	2 (4.3)	14 (35.9)	3 (12.5)	19 (36.5)	
Time to metastasis (month, mean ± SD)	30.3 ± 38.0	29.3 ± 29.2	32.7 ± 32.6	18.2 ± 16.8	35.1 ± 38.0	0.182
Patients death	53 (32.7)	23 (48.9)	11 (28.2)	7 (29.2)	12 (23.1)	**0.040**

Bold represents p < 0.05.

### Expression of metabolism-related proteins in breast cancer metastasis according to metastatic site (Figure 1)

**Figure 1:**
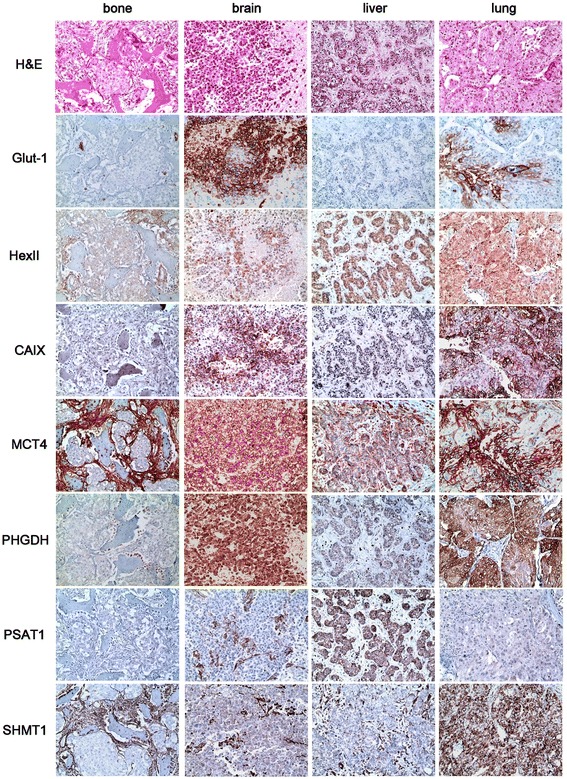
**Differential expression of metabolism-related proteins in breast cancer metastasis according to metastatic site.** The expression of glycolysis-related proteins (Glut-1, hexokinase II, CAIX, and MCT4) was higher in the brain and lower in the bone and liver. The stromal expression levels of MCT4, PHGDH, and SHMT1 were higher in bone metastasis than other sites.

Analysis of metabolism-related protein expression according to metastatic site in metastatic breast cancer revealed site-specific expression patterns of glycolysis-related proteins (Glut-1, hexokinase II, CAIX, and MCT4), with higher expression levels seen in brain metastasis than in bone or liver metastasis (p < 0.001, 0.001, 0.009, and <0.001, respectively). Similar trends were seen for PHGDH (p = 0.027). The highest expression levels of SDHB and SHMT1 were found in lung metastasis, while the lowest levels were seen in bone metastasis (p = 0.006, and 0.033, respectively) (Table 3).

**Table 3:** **Expression of metabolism-related proteins in the tumor cell compartment of breast cancer metastasis according to metastatic site**

**Parameters**	**Total N = 162 (%)**	**Bone metastasis n = 47 (%)**	**Brain metastasis n = 39 (%)**	**Liver metastasis n = 24 (%)**	**Lung metastasis n = 52 (%)**	**p-value**
Glut-1						**<0.001**
Negative	83 (51.2)	35 (74.5)	10 (25.6)	18 (75.0)	20 (38.5)	
Positive	79 (48.8)	12 (25.5)	29 (74.4)	6 (25.0)	32 (61.5)	
Hexokinase II						**0.001**
Negative	113 (69.8)	41 (87.2)	25 (64.1)	20 (83.3)	27 (51.9)	
Positive	49 (30.2)	6 (12.8)	14 (35.9)	4 (16.7)	25 (48.1)	
CAIX						**0.009**
Negative	130 (80.2)	44 (93.6)	26 (66.7)	21 (87.5)	39 (75.0)	
Positive	32 (19.8)	3 (6.4)	13 (33.3)	3 (12.5)	13 (25.0)	
MCT4						**<0.001**
Negative	66 (40.7)	25 (53.2)	4 (10.3)	13 (54.2)	24 (46.2)	
Positive	96 (59.3)	22 (46.8)	35 (89.7)	11 (45.8)	28 (53.8)	
GLS1						0.473
Negative	83 (51.2)	28 (59.6)	17 (43.6)	11 (45.8)	27 (51.9)	
Positive	79 (48.8)	19 (40.4)	22 (56.4)	13 (54.2)	25 (48.1)	
GDH						0.610
Negative	2 (1.2)	1 (2.1)	1 (2.6)	0 (0.0)	0 (0.0)	
Positive	160 (98.8)	46 (97.9)	38 (97.4)	24 (100.0)	52 (100.0)	
ASCT2						**0.033**
Negative	122 (75.3)	37 (78.7)	34 (87.2)	19 (79.2)	32 (61.5)	
Positive	40 (24.7)	10 (21.3)	5 (12.8)	5 (20.8)	20 (38.5)	
ATP synthase						0.610
Negative	2 (1.2)	1 (2.1)	1 (2.6)	0 (0.0)	0 (0.0)	
Positive	160 (98.8)	46 (97.9)	38 (97.4)	24 (100.0)	52 (100.0)	
SDHA						0.175
Negative	2 (1.2)	2 (4.3)	0 (0.0)	0 (0.0)	0 (0.0)	
Positive	160 (98.8)	45 (95.7)	39 (100.0)	24 (100.0)	52 (100.0)	
SDHB						**0.006**
Negative	52 (32.1)	20 (42.6)	16 (41.0)	9 (37.5)	7 (13.5)	
Positive	110 (67.9)	27 (57.4)	23 (59.0)	15 (62.5)	45 (86.5)	
PHGDH						**0.027**
Negative	61 (37.7)	24 (51.1)	11 (28.2)	12 (50.0)	14 (26.9)	
Positive	101 (62.3)	23 (48.9)	28 (71.8)	12 (50.0)	38 (73.1)	
PSPH						0.926
Negative	146 (90.1)	42 (89.4)	36 (92.3)	22 (91.7)	46 (88.5)	
Positive	16 (9.9)	5 (10.6)	3 (7.7)	2 (8.3)	6 (11.5)	
PSAT1						**<0.001**
Negative	140 (86.4)	41 (87.2)	37 (94.9)	14 (58.3)	48 (92.3)	
Positive	22 (13.6)	6 (12.8)	2 (5.1)	10 (41.7)	4 (7.7)	
SHMT1						**0.033**
Negative	127 (78.4)	43 (91.5)	31 (79.5)	18 (75.0)	35 (67.3)	
Positive	35 (21.6)	4 (8.5)	8 (20.5)	6 (25.0)	17 (32.7)	
GLDC						0.547
Negative	96 (59.3)	31 (66.0)	24 (61.5)	14 (58.3)	27 (51.9)	
Positive	66 (40.7)	16 (34.0)	15 (38.5)	10 (41.7)	25 (48.1)	

Bold represents p < 0.05.

On analysis of metabolism-related protein expression in the stromal compartment of tumors, the expression of MCT4 (p = 0.002), GLS1 (p = 0.006), GDH (p = 0.035), SDHA (p = 0.004), PHGDH (p = 0.018), PSPH (p = 0.006), PSAT1 (p < 0.001), and SHMT1 (p < 0.001) showed site specificity: higher stromal expression of MCT4, GLS1, GDH, SDHA, PHGDH, and SHMT1 were found in bone metastasis, while PSPH and PSAT1 were higher in brain metastasis (Table 4).

**Table 4:** **Expression of metabolism-related proteins in the stromal compartment of breast cancer metastasis according to metastatic site**

**Parameters**	**Total N = 162 (%)**	**Bone metastasis n = 47 (%)**	**Brain metastasis n = 39 (%)**	**Liver metastasis n = 24 (%)**	**Lung metastasis n = 52 (%)**	**p-value**
Hexokinase II						0.058
Negative	159 (98.1)	44 (93.6)	39 (100.0)	24 (100.0)	52 (100.0)	
Positive	3 (1.9)	3 (6.4)	0 (0.0)	0 (0.0)	0 (0.0)	
MCT4						**0.002**
Negative	113 (69.8)	26 (55.3)	36 (92.3)	17 (70.8)	34 (65.4)	
Positive	49 (30.2)	21 (44.7)	3 (7.7)	7 (29.2)	18 (34.6)	
GLS1						**0.006**
Negative	157 (96.9)	42 (89.4)	39 (100.0)	24 (100.0)	52 (100.0)	
Positive	5 (3.1)	5 (10.6)	0 (0.0)	0 (0.0)	0 (0.0)	
GDH						**0.035**
Negative	140 (86.4)	36 (76.6)	38 (97.4)	22 (91.7)	44 (84.6)	
Positive	22 (13.6)	11 (23.4)	1 (2.6)	2 (8.3)	8 (15.4)	
ATP synthase						0.084
Negative	155 (95.7)	42 (89.4)	38 (97.4)	24 (100.0)	51 (98.1)	
Positive	7 (4.3)	5 (10.6)	1 (2.6)	0 (0.0)	1 (1.9)	
SDHA						**0.004**
Negative	145 (89.5)	36 (76.6)	38 (97.4)	21 (87.5)	50 (96.2)	
Positive	17 (10.5)	11 (23.4)	1 (2.6)	3 (12.5)	2 (3.8)	
PHGDH						**0.018**
Negative	158 (97.5)	43 (91.5)	39 (100.0)	24 (100.0)	52 (100.0)	
Positive	4 (2.5)	4 (8.5)	0 (0.0)	0 (0.0)	0 (0.0)	
PSPH						**0.006**
Negative	156 (96.3)	46 (97.9)	34 (87.2)	24 (100.0)	52 (100.0)	
Positive	6 (3.7)	1 (2.1)	5 (12.8)	0 (0.0)	0 (0.0)	
PSAT1						**<0.001**
Negative	154 (95.1)	46 (97.9)	32 (82.1)	24 (100.0)	52 (100.0)	
Positive	8 (4.9)	1 (2.1)	7 (17.9)	0 (0.0)	0 (0.0)	
SHMT1						**<0.001**
Negative	98 (60.5)	16 (34.0)	31 (79.5)	16 (66.7)	35 (67.3)	
Positive	64 (39.5)	31 (66.0)	8 (20.5)	8 (33.3)	17 (32.7)	

Bold represents p < 0.05.

After a review of the metabolic phenotype according to metastatic site, the glycolysis phenotype was most often seen in the brain and lung (p < 0.001) (Table 5).

**Table 5:** **Metabolic phenotypes of breast cancer metastasis according to metastatic site**

**Parameters**	**Total N = 162 (%)**	**Bone metastasis n = 47 (%)**	**Brain metastasis n = 39 (%)**	**Liver metastasis n = 24 (%)**	**Lung metastasis n = 52 (%)**	**p-value**
Glycolysis type						**<0.001**
No	81 (50.0)	36 (76.6)	9 (23.1)	17 (70.8)	19 (36.5)	
Yes	81 (50.0)	11 (23.4)	30 (76.9)	7 (29.2)	33 (63.5)	
Glutamine type						0.308
No	62 (38.4)	23 (48.9)	14 (35.9)	9 (37.5)	16 (30.8)	
Yes	100 (61.7)	24 (51.1)	25 (64.1)	15 (62.5)	36 (69.2)	
Mitochondrial type						0.175
No	2 (1.2)	2 (4.3)	0 (0.0)	0 (0.0)	0 (0.0)	
Yes	160 (98.8)	45 (95.7)	39 (100.0)	24 (100.0)	52 (100.0)	
Serine/glycine type						0.444
No	128 (79.0)	37 (78.7)	34 (87.2)	19 (79.2)	38 (73.1)	
Yes	34 (21.0)	10 (21.3)	5 (12.8)	5 (20.8)	14 (26.9)	

Bold represents p < 0.05.

### Correlation of expression of metabolism-related proteins between primary and metastatic breast cancer according to metastatic site

**Figure 2:**
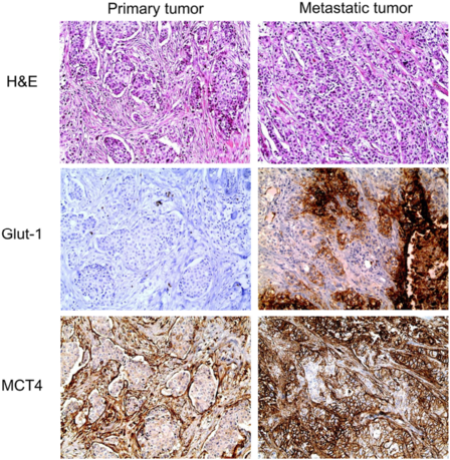
**Expression of Glut-1 and MCT4 in primary and metastatic breast cancer.** There was no expression of Glut-1 and MCT4 in primary breast cancer, while the expression of Glut-1 and MCT4 increased in lung metastasis.

We analyzed the expression levels of metabolism-related proteins in primary and metastatic cancers in 49 paired cases. The expression level of MCT4 was statistically different between primary and metastatic cancers (p = 0.002). When considering difference between primary and metastatic cancers according to metastatic sites, Glut-1 (p = 0.004) and MCT4 (p = 0.004) were expressed in the lung metastasis but not in the primary cancer (Table 6 and Figure 2).

**Table 6:** **Correlation of expression of metabolism related proteins between primary and metastatic breast cancer according to metastatic site**

**Parameters**	**Total**		**Bone metastasis**		**Brain metastasis**		**Liver metastasis**		**Lung metastasis**	
	**N = 49 (%)**	**p-value**	**n = 13 (%)**	**p-value**	**n = 9 (%)**	**p-value**	**n = 4 (%)**	**p-value**	**n = 23 (%)**	**p-value**
Glut-1		0.481		0.063		1.000		1.000		**0.004**
(+) → (+)	10 (20.4)		0 (0.0)		4 (44.4)		0 (0.0)		6 (20.4)	
(+) → (−)	7 (14.3)		5 (38.5)		1 (11.1)		1 (25.0)		0 (0.0)	
(−) → (+)	11 (22.4)		0 (0.0)		2 (22.2)		0 (0.0)		9 (39.1)	
(−) → (−)	21 (42.9)		8 (61.5)		2 (22.2)		3 (75.0)		8 (34.8)	
Hexokinase II		0.581		1.000		0.500		1.000		1.000
(+) → (+)	11 (22.4)		1 (7.7)		1 (11.1)		0 (0.0)		9 (39.1)	
(+) → (−)	5 (10.2)		3 (23.1)		0 (0.0)		0 (0.0)		2 (8.7)	
(−) → (+)	8 (16.3)		2 (15.4)		2 (22.2)		1 (25.0)		3 (13.0)	
(−) → (−)	25 (51.0)		7 (53.8)		6 (66.7)		3 (75.0)		9 (39.1)	
CAIX		0.688		N/A		1.000		N/A		1.000
(+) → (+)	2 (4.1)		0 (0.0)		0 (0.0)		0 (0.0)		2 (8.7)	
(+) → (−)	2 (4.1)		0 (0.0)		1 (11.1)		0 (0.0)		1 (4.3)	
(−) → (+)	4 (8.2)		0 (0.0)		2 (22.2)		0 (0.0)		2 (8.7)	
(−) → (−)	41 (83.7)		13 (100.0)		6 (66.7)		4 (100.0)		18 (78.3)	
MCT4		**0.002**		0.250		0.250		0.500		**0.004**
(+) → (+)	14 (28.6)		3 (23.1)		4 (44.4)		0 (0.0)		7 (30.4)	
(+) → (−)	2 (4.1)		0 (0.0)		0 (0.0)		2 (50.0)		0 (0.0)	
(−) → (+)	15 (30.6)		3 (23.1)		3 (33.3)		0 (0.0)		9 (39.1)	
(−) → (−)	18 (36.7)		7 (53.8)		2 (22.2)		2 (50.0)		7 (30.4)	
GLS1		1.000		0.063		1.000		1.000		0.289
(+) → (+)	15 (30.6)		2 (15.4)		3 (33.3)		2 (50.0)		8 (34.8)	
(+) → (−)	8 (16.3)		0 (0.0)		1 (11.1)		1 (25.0)		6 (26.1)	
(−) → (+)	9 (18.4)		5 (38.5)		2 (22.2)		0 (0.0)		2 (8.7)	
(−) → (−)	17 (34.7)		6 (46.2)		3 (33.3)		1 (25.0)		7 (30.4)	
GDH		1.000		1.000		1.000		1.000		1.000
(+) → (+)	44 (89.8)		11 (84.6)		8 (88.9)		3 (75.0)		22 (95.7)	
(+) → (−)	2 (4.1)		1 (7.7)		1 (11.1)		0 (0.0)		0 (0.0)	
(−) → (+)	3 (6.1)		1 (7.7)		0 (0.0)		1 (25.0)		1 (4.3)	
(−) → (−)	0 (0.0)		0 (0.0)		0 (0.0)		0 (0.0)		0 (0.0)	
ASCT2		0.092		1.000		0.219		1.000		0.375
(+) → (+)	8 (16.3)		0 (0.0)		0 (0.0)		1 (25.0)		7 (30.4)	
(+) → (−)	10 (20.4)		1 (7.7)		5 (55.6)		0 (0.0)		4 (17.4)	
(−) → (+)	3 (6.1)		1 (7.7)		1 (11.1)		0 (0.0)		1 (4.3)	
(−) → (−)	28 (57.1)		11 (84.6)		3 (33.3)		3 (75.0)		11 (47.8)	
ATP synthase		1.000		1.000		N/A		N/A		N/A
(+) → (+)	48 (98.0)		12 (92.3)		9 (100.0)		4 (100.0)		23 (100.0)	
(+) → (−)	1 (2.0)		1 (7.7)		0 (0.0)		0 (0.0)		0 (0.0)	
(−) → (+)	0 (0.0)		0 (0.0)		0 (0.0)		0 (0.0)		0 (0.0)	
(−) → (−)	0 (0.0)		0 (0.0)		0 (0.0)		0 (0.0)		0 (0.0)	
SDHA		1.000		1.000		1.000		N/A		N/A
(+) → (+)	44 (89.8)		9 (69.2)		8 (88.9)		4 (100.0)		23 (100.0)	
(+) → (−)	2 (4.1)		2 (15.4)		0 (0.0)		0 (0.0)		0 (0.0)	
(−) → (+)	3 (6.1)		2 (15.4)		1 (11.1)		0 (0.0)		0 (0.0)	
(−) → (−)	0 (0.0)		0 (0.0)		0 (0.0)		0 (0.0)		0 (0.0)	
SDHB		0.263		1.000		1.000		1.000		0.063
(+) → (+)	24 (49.0)		4 (30.8)		3 (33.3)		1 (25.0)		16 (69.9)	
(+) → (−)	7 (14.3)		3 (23.1)		3 (33.3)		1 (25.0)		0 (0.0)	
(−) → (+)	13 (26.5)		3 (23.1)		3 (33.3)		2 (50.0)		5 (21.7)	
(−) → (−)	5 (10.2)		3 (23.1)		0 (0.0)		0 (0.0)		2 (8.7)	
PHGDH		0.581		1.000		1.000		1.000		0.688
(+) → (+)	22 (44.9)		3 (23.1)		6 (66.7)		1 (25.0)		12 (52.2)	
(+) → (−)	5 (10.2)		2 (15.4)		1 (11.1)		0 (0.0)		2 (8.7)	
(−) → (+)	8 (16.3)		2 (15.4)		1 (11.1)		1 (25.0)		4 (17.4)	
(−) → (−)	14 (28.6)		6 (46.2)		1 (11.1)		2 (50.0)		5 (21.7)	
PSPH		1.000		N/A		1.000		1.000		1.000
(+) → (+)	1 (2.0)		0 (0.0)		0 (0.0)		0 (0.0)		1 (4.3)	
(+) → (−)	3 (6.1)		0 (0.0)		1 (11.1)		0 (0.0)		2 (8.7)	
(−) → (+)	2 (4.1)		0 (0.0)		0 (0.0)		1 (25.0)		1 (4.3)	
(−) → (−)	43 (87.8)		13 (100.0)		8 (88.9)		3 (75.0)		19 (82.6)	
PSAT1		0.607		1.000		1.000		1.000		0.219
(+) → (+)	1 (2.0)		0 (0.0)		0 (0.0)		0 (0.0)		1 (4.3)	
(+) → (−)	9 (18.4)		3 (23.1)		0 (0.0)		1 (25.0)		5 (21.7)	
(−) → (+)	6 (12.2)		2 (15.4)		1 (11.1)		2 (50.0)		1 (4.3)	
(−) → (−)	33 (67.3)		8 (61.5)		8 (88.9)		1 (25.0)		16 (69.6)	
SHMT1		1.000		1.000		1.000		N/A		1.000
(+) → (+)	10 (20.4)		0 (0.0)		2 (22.2)		0 (0.0)		8 (34.8)	
(+) → (−)	2 (4.1)		1 (7.7)		0 (0.0)		0 (0.0)		1 (4.3)	
(−) → (+)	1 (2.0)		0 (0.0)		0 (0.0)		0 (0.0)		1 (4.3)	
(−) → (−)	36 (73.5)		12 (92.3)		7 (77.8)		4 (100.0)		13 (56.5)	
GLDC		0.143		0.375		1.000		1.000		0.508
(+) → (+)	12 (24.5)		0 (0.0)		3 (33.3)		1 (25.0)		8 (34.8)	
(+) → (−)	5 (10.2)		1 (7.7)		1 (11.1)		0 (0.0)		3 (13.0)	
(−) → (+)	12 (24.5)		4 (30.8)		1 (11.1)		1 (25.0)		6 (26.1)	
(−) → (−)	20 (40.8)		8 (61.5)		4 (44.4)		2 (50.0)		6 (26.1)	

*p-value was calculated by McNemar test.

Bold represents p < 0.05.

### Correlation between pathologic factors and expression of metabolism-related proteins

**Figure 3:**
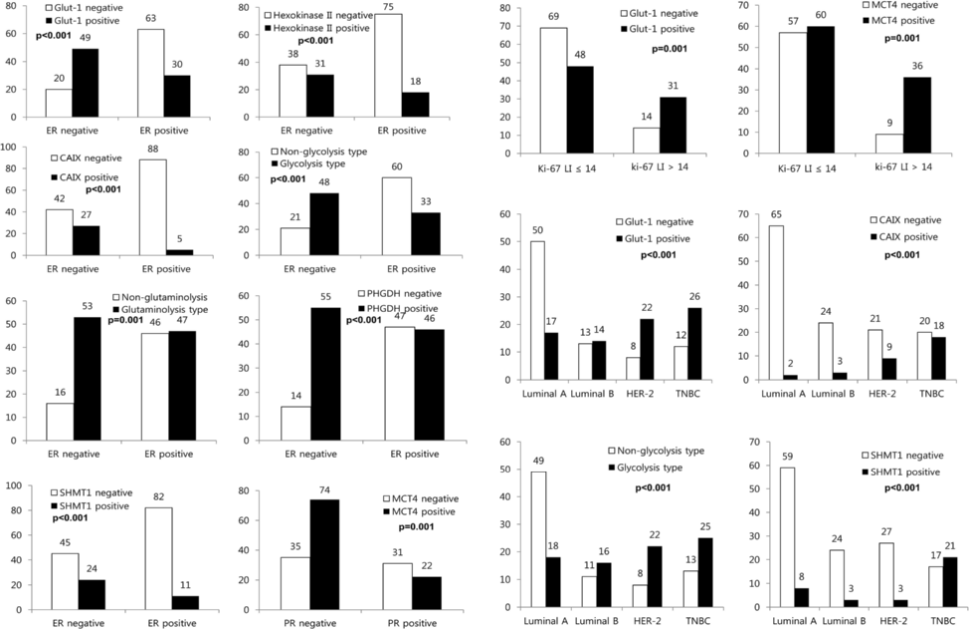
**Correlation between pathologic factors and expression of metabolism-related proteins.**

On analyzing the association between expression of metabolism-related proteins and pathologic factors, ER negativity was associated with Glut-1 positivity (p < 0.001), hexokinase II positivity (p < 0.001), CAIX positivity (p < 0.001), glycolysis type (p < 0.001), glutaminolysis type (p = 0.001), PHGDH positivity (p < 0.001), and SHMT1 positivity (p < 0.001). PR negativity was associated with MCT4 positivity (p = 0.001) and higher Ki-67 LI was associated with Glut-1 positivity (p = 0.001) and MCT4 positivity (p = 0.001). Glut-1 (p < 0.001), CAIX (p < 0.001), and SHMT1 (p < 0.001) were associated with molecular subtype. If these proteins were expressed, the proportion of TNBC was higher, while luminal A type was higher when these proteins were not expressed. In addition, TNBC was more common in glycolysis type, while luminal A was more common in non-glycolysis type (p < 0.001) (Figure 3).

### The association between the expression of metabolism-related proteins and patient prognosis

**Figure 4:**
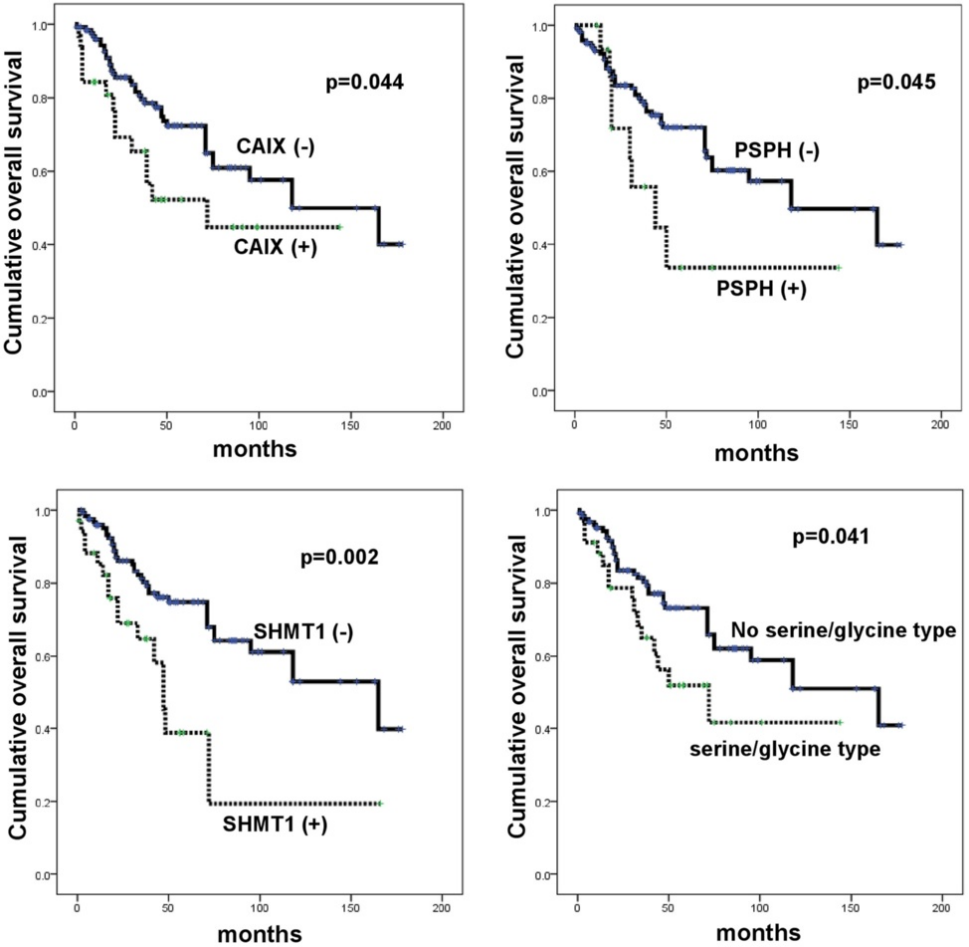
**Association between expression level of metabolism-related proteins and patient prognosis in metastatic breast cancer.**

On analyzing the association of metabolic phenotype and the expression of metabolism-related proteins with patient prognosis with univariate analysis (Figure 4 and Table 7), we found that factors associated with shorter overall survival (OS) were CAIX positivity (p = 0.044), PSPH positivity (p = 0.045), SHMT1 positivity (p = 0.002), and serine/glycine type (p = 0.041). The factors associated with shorter OS in multivariate analysis were higher Ki-67 LI (hazard ratio: 4.096, 95% CI: 1.664–10.08, P = 0.002) and tumoral SHMT1 positivity (hazard ratio: 2.836, 95% CI: 1.239–6.495, P = 0.014) (Table 8).

**Table 7:** **Univariate analysis of the association between expression levels of metabolism-related proteins in metastatic breast cancers and overall survival by the log-rank test**

**Parameters**	**Total N = 162 (%)**		**Bone metastasis n = 47 (%)**		**Brain metastasis n = 39 (%)**		**Liver metastasis n = 24 (%)**		**Lung metastasis n = 52 (%)**	
	**Mean survival (95% CI) months**	***P*** **-value**	**Mean survival (95% CI) months**	***P*** **-value**	**Mean survival (95% CI) months**	***P*** **-value**	**Mean survival (95% CI) months**	***P*** **-value**	**Mean survival (95% CI) months**	***P*** **-value**
Glut-1		0.141		**0.020**		0.504		0.591		0.833
Negative	121 (103–139)		102 (76–129)		94 (65–123)		84 (64–105)		131 (97–165)	
Positive	92 (72–111)		56 (41–70)		101 (74–127)		62 (35–88)		120 (89–150)	
Hexokinase II		0.727		0.912		0.680		0.418		0.192
Negative	112 (96–128)		83 (61–104)		103 (76–130)		85 (65–105)		146 (117–175)	
Positive	96 (68–124)		62 (42–81)		54 (42–65)		56 (21–90)		103 (69–137)	
CAIX		**0.044**		**<0.001**		0.527		0.964		**0.046**
Negative	115 (100–130)		87 (67–108)		115 (90–141)		81 (61–101)		140 (114–166)	
Positive	80 (56–103)		16 (0–41)		64 (42–86)		67 (30–105)		87 (49–124)	
MCT4		0.612		0.787		0.995		0.652		0.456
Negative	107 (87–128)		92 (62–122)		73 (29–117)		85 (63–107)		120 (85–154)	
Positive	113 (95–131)		66 (55–77)		107 (84–130)		61 (40–82)		138 (107–168)	
GLS1		0.274		0.690		0.061		0.348		0.896
Negative	114 (96–132)		85 (61–108)		133 (108–158)		70 (52–88)		127 (95–160)	
Positive	110 (91–130)		61 (43–78)		61 (43–80)		72 (47–98)		133 (102–164)	
GDH		0.919		0.171		n/a		n/a		n/a
Negative	165 (165–165)		165 (165–165)		n/a		n/a		n/a	
Positive	111 (97–125)		72 (58–86)		n/a		n/a		n/a	
ASCT2		0.686		0.948		n/a		0.761		0.730
Negative	111 (96–127)		89 (64–114)		n/a		83 (62–104)		135 (107–162)	
Positive	89 (74–104)		80 (47–114)		n/a		58 (40–76)		70 (57–83)	
ATP synthase		0.965		0.171		n/a		n/a		n/a
Negative	165 (165–165)		165 (165–165)		n/a		n/a		n/a	
Positive	111 (97–125)		72 (58–86)		n/a		n/a		n/a	
SDHA		0.830		0.132		n/a		n/a		n/a
Negative	165 (165–165)		165 (165–165)		n/a		n/a		n/a	
Positive	111 (96–125)		72 (57–86)		n/a		n/a		n/a	
SDHB		0.460		0.630		0.372		0.870		0.649
Negative	102 (80–124)		85 (52–118)		115 (85–146)		49 (45–53)		82 (48–115)	
Positive	117 (101–133)		68 (57–80)		70 (52–88)		80 (57–103)		135 (112–158)	
PHGDH		0.590		0.494		0.878		**0.048**		0.939
Negative	117 (96–138)		90 (61–119)		84 (53–115)		78 (65–90)		127 (80–174)	
Positive	108 (90–126)		60 (48–71)		107 (81–133)		64 (39–89)		122 (89–154)	
PSPH		**0.045**		**0.011**		0.714		0.654		0.477
Negative	114 (99–128)		88 (67–110)		108 (86–131)		83 (63–102)		131 (105–157)	
Positive	68 (36–100)		35 (22–49)		30 (17–42)		53 (22–83)		94 (41–147)	
PSAT1		0.542		n/a		n/a		0.927		0.074
Negative	109 (94–123)		n/a		n/a		83 (59–106)		134 (109–158)	
Positive	79 (63–95)		n/a		n/a		65 (46–83)		38 (11–65)	
SHMT1		**0.002**		0.258		**0.022**		0.089		**0.009**
Negative	119 (104–134)		84 (64–105)		117 (95–140)		90 (71–109)		147 (121–172)	
Positive	63 (31–94)		31 (7–56)		27 (19–34)		39 (24–55)		78 (32–123)	
GLDC		0.281		0.485		**0.024**		0.281		0.370
Negative	115 (98–133)		80 (55–104)		127 (105–150)		89 (68–111)		135 (102–168)	
Positive	99 (80–119)		66 (56–76)		53 (31–76)		59 (35–82)		111 (78–144)	
Glycolysis type		0.615		0.159		0.614		0.921		0.961
No	116 (97–134)		96 (71–122)		91 (59–123)		82 (61–103)		128 (91–164)	
Yes	99 (80–118)		63 (51–75)		102 (76–128)		69 (44–94)		123 (95–151)	
Glutamine type		0.116		0.661		0.251		0.213		0.454
No	121 (101–142)		90 (59–120)		128 (96–159)		74 (54–93)		140 (105–175)	
Yes	99 (79–119)		72 (51–93)		68 (51–84)		72 (49–95)		129 (101–157)	
Mitochondrial type		0.830		0.132		n/a		n/a		n/a
No	165 (165–165)		165 (165–165)		n/a		n/a		n/a	
Yes	111 (96–125)		72 (57–86)		n/a		n/a		n/a	
Serine/glycine type		**0.041**		0.886		0.467		**0.034**		**0.019**
No	116 (101–131)		83 (61–105)		109 (86–133)		90 (72–109)		142 (116–168)	
Yes	79 (56–103)		60 (45–76)		66 (30–103)		38 (15–60)		72 (32–112)	
Molecular subtypes		**0.002**		**<0.001**		0.081		N/A		N/A
Luminal A	105 (86–124)		84 (62–107)		55 (10–100)		N/A		N/A	
Luminal B	140 (111–170)		60 (26–93)		138 (112–164)		N/A		N/A	
HER-2	134 (109–158)		62 (47–77)		79 (60–97)		N/A		N/A	
TNBC	51 (38–64)		3 (2–4)		31 (22–39)		N/A		N/A	

Bold represents p < 0.05.

**Table 8:** **Multivariate analysis of patient prognosis in metastatic breast cancer**

**Parameters**	**Overall survival**		
	**Hazard ratio**	**95% CI**	***P*** **-value**
ER status			0.067
Negative versus positive	10.42	0.846-128.4	
PR status			0.091
Negative versus positive	1.195	0.898-4.237	
HER2 status			0.408
Negative versus positive	0.436	0.075-2.869	
Ki-67 LI			**0.002**
≤14 versus >14	4.096	1.664-10.08	
Tumor phenotypes			0.147
Luminal A			
Luminal B	6.697	0.387-116.0	
HER2	7.286	0.348-152.4	
TNBC	0.433	0.055-3.387	
CAIX			0.189
Negative versus positive	1.690	0.773-3.695	
PSPH			0.117
Negative versus positive	2.156	0.825-5.634	
SHMT1			**0.014**
Negative versus positive	2.836	1.239-6.495	
Serine/glycine type			0.451
No versus Yes	0.723	0.311-1.679	

Bold represents p < 0.05.

**Figure 5:**
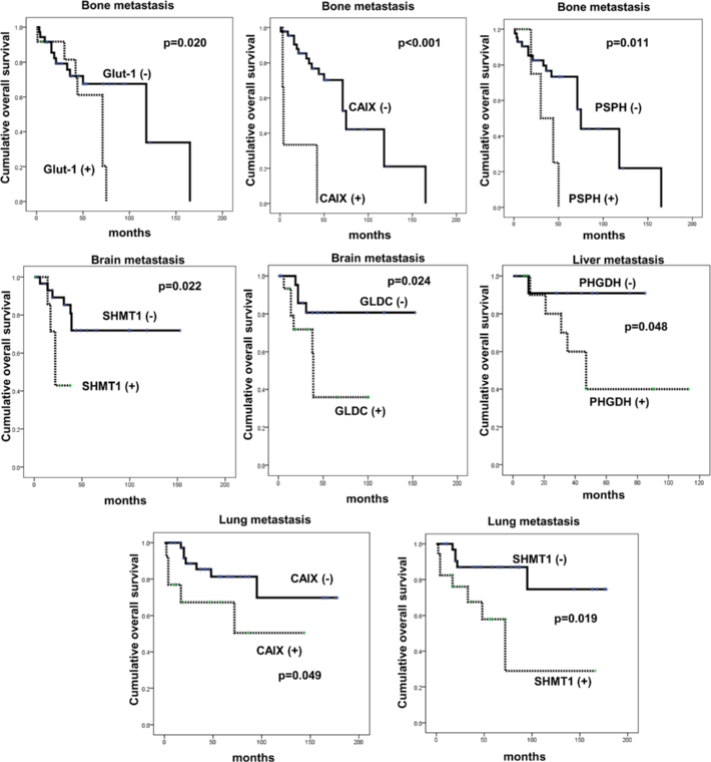
**Association between expression level of metabolism-related proteins and patient prognosis in metastatic breast cancer according to the metastatic sites.**

Univariate analysis was performed to analyze the association between expression of metabolism-related proteins and metabolic phenotype according to metastatic site. The factors associated with shorter OS were Glut-1 positivity (p = 0.020), CAIX positivity (p < 0.001), and PSPH positivity (p = 0.011) in bone metastasis. SHMT1 positivity (p = 0.022) and GLDC positivity (p = 0.024) were associated with shorter OS in brain metastasis, PHGDH positivity (p = 0.048) was associated with shorter OS in liver metastasis, and CAIX positivity (p = 0.046) was associated with shorter OS in lung metastasis (Figure 5 and Table 7).

## Discussion

In this study, differences in metabolic features of tumors were seen according to metastatic site among cases of metastatic breast cancer. Briefly, brain and lung metastasis showed higher expression levels of glycolysis-related proteins (Glut-1, hexokinase II, CAIX, and MCT4) than did bone and liver metastasis. Thus, there are several possible reasons for the different metabolic features according to metastatic site. First, the molecular subtypes showed site specificity in metastatic breast cancer, with a high proportion of TNBC in brain and lung metastases and luminal A in bone and liver metastases. In previous studies, the expression levels of glycolysis-related proteins were higher in TNBC or basal-like type [25,26]. Such data are in agreement with the results from this study that the expression of glycolysis-related proteins is higher in brain and lung metastases, which consist of more TNBC cases. Another possible reason is the variety of influences from circulation tumor cells (CTC). Given that CTC, defined as cancer cells in the blood of cancer patients, plays a significant role in the metastatic process, CTC and its metabolites have an influence on metastatic properties. This may result in site specificity according to metastatic site; however, further study is required [27]. One other reason is the different metabolic characteristics of metastatic sites. For example, given that bone tissue creates the hematopoietic cells, the metabolites of bone tissue are expected to differ from those of liver, brain, or lung. This is supported by the fact that immune-responsive tissue and immune-privileged tissues are reported to show different cellular compositions, as well as different metabolic and immunological responses [28].

The stromal expression levels of hexokinase II, MCT4, GLS1, GDH, SDHA, PHGDH, and SHMT1 were higher in bone metastasis compared to that at other sites. In terms of histologic features, bone metastasis forms highly rich tumor stroma with prominent fibroblasts. The reverse-Warburg effect theory, which describes the metabolic interaction between tumor cells and the stroma, supports the expression of metabolism-related proteins in the stroma in bone metastasis. The theory insists that lactate created by glycolysis in the stroma is transferred to tumor cells and metabolized as the substrate by oxidative phosphorylation in tumor cells [29,30]. Therefore, the reverse Warburg effect phenotype may be applicable to bone metastasis, in which glycolysis-related molecules or glycolytic metabolism intermediates are highly expressed. In previous studies, luminal type tumors were more likely to have the reverse Warburg effect phenotype [31]. This may explain why metastatic tumors with the reverse Warburg effect phenotype are more likely to occur in the bone, since luminal type tumors are most commonly found in the bone. However, further validation studies are needed to confirm these findings.

Primary tumors were negative for Glut-1 and MCT4, but some positivity was seen in lung metastasis. Previous studies reported differential expression of most important biomarkers of breast cancer (ER, PR, and HER-2) between primary cancer and metastatic cancer, with 21–50% showing HER-2 loss, about 30% showing HER-2 gain [8,32], 3.2–44% showing ER loss [33-35], 24% showing PR loss [35], and ER or PR gain not reported. In other words, when primary breast cancer progresses to metastatic cancer, expression of ER/PR can be lost. The loss of ER/PR may be because metastatic cancer exhibits more aggressive features compared to primary tumors; thus, ER/PR, a good prognostic marker, presents as a loss rather than as a gain. In this study, Glut-1 expression was associated with ER negativity and MCT4 expression was associated with PR negativity. In the progression of primary to metastatic cancer, ER/PR is lost and the expression of metabolism-related proteins like Glut-1 and MCT4 appear. Further validation is required to generalize the findings of this study.

The clinical significance of this study is that inhibition of the metabolic pathway may be a potential treatment target. The expression of metabolism-related proteins, especially glycolysis-related proteins differed according to metastatic site. Previous preclinical studies reported that Glut-1 inhibitor [36,37], CAIX inhibitor [38], and MCT4 inhibitor [39] suppress tumor growth in several tumor types. Thus, these proteins are possible targets for chemotherapy in brain and lung metastasis, which showed higher expression levels of glycolysis-related proteins. However, it should be noted that a compensating response may appear if one or two molecules are inhibited in metabolic pathway targeted therapy [40].

## Conclusion

In conclusion, differences in metabolic features according to metastatic site were seen in metastatic breast cancer, with the glycolysis phenotype found predominantly in the brain and lung since the expression of glycolysis-related protein was higher and the non-glycolysis phenotype in the bone and liver.

## Abbreviations

ER: Estrogen receptor
PR: Progesterone receptor
FFPE: Formalin-fixed, paraffin-embedded
IHC: Immunohistochemistry
ASCO: American Society of Clinical Oncology
CAP: College of American Pathologists
TNBC: Triple negative breast cancer
PHGDH: Phosphoglycerate dehydrogenase
PSAT: Phosphoserine aminotransferase
PSPH: Phosphoserine phosphatase
GLDC: Glycine decarboxylase
SHMT: Serine hydroxymethyltransferase
CAIX: Carbonic anhydrase
MCT: Monocarboxylate transporters
GLS: Glutaminase
GDH: Glutamate dehydrogenase
ASCT: Amino acid transporter
SDH: Succinate dehydrogenase
